# Association Between Triglyceride‐Glucose Index and Breast Cancer: A Systematic Review and Meta‐Analysis

**DOI:** 10.1002/cnr2.70194

**Published:** 2025-04-07

**Authors:** Diar Zooravar, Hanieh Radkhah, Bahareh Shateri Amiri, Pedram Soltani

**Affiliations:** ^1^ School of Medicine Iran University of Medical Sciences Tehran Iran; ^2^ Department of Internal Medicine, School of Medicine Sina Hospital, Tehran University of Medical Sciences Tehran Iran; ^3^ Department of Internal Medicine, School of Medicine Hazrat‐e‐Rasool General Hospital, Iran University of Medical Sciences Tehran Iran

**Keywords:** breast cancer, cancer screening, insulin resistance, metabolic syndrome, triglyceride‐glucose index, TyG index

## Abstract

**Background and Aims:**

The triglyceride‐glucose (TyG) index, a surrogate marker for insulin resistance and metabolic syndrome, has been implicated in breast cancer (BC) risk. However, its predictive value remains controversial. This systematic review and meta‐analysis assessed the association between the TyG index and BC risk, its role in differentiating malignant from benign breast lesions, and its potential prognostic significance.

**Methods:**

A comprehensive search of PubMed, Scopus, Web of Science, Embase, and Google Scholar was conducted up to January 2025. Studies were included if they met the following criteria: (1) assessed the TyG index about BC risk, progression, or prognosis; (2) included a comparator group (healthy individuals, benign breast lesion patients, or internal controls); (3) reported effect sizes (odds ratio [OR] or hazard ratio [HR]) with 95% confidence intervals (CI); and (4) provided sufficient statistical data on the TyG index. Excluded studies included in vitro or animal research, reviews, case reports, and those lacking relevant quantitative data. Effect sizes were pooled using a random‐effects model; heterogeneity was assessed via *I* ^2^ statistics, and sensitivity analyses were performed. A restricted cubic spline model evaluated dose–response relationships.

**Results:**

Thirteen studies, including retrospective, case–control, cohort, and cross‐sectional designs, were analyzed. Case–control and cross‐sectional studies revealed a significant association between a higher TyG index and increased BC risk (OR: 1.87, 95% CI: 1.45–2.41, *p* < 0.01). However, cohort studies did not confirm this relationship (HR: 1.04, 95% CI: 0.97–1.11, *p* = 0.23). The TyG index effectively differentiated malignant from benign breast lesions (WMD: 0.23, 95% CI: 0.18–0.27, *p* < 0.01) with a pooled AUC of 0.64. Dose–response analysis suggested a non‐linear relationship between the TyG index and BC risk (*p* < 0.001).

**Conclusion:**

While the TyG index may not strongly predict BC onset, it reflects metabolic alterations linked to cancer progression. Its ability to distinguish benign from malignant lesions highlights its clinical utility. Future studies should standardize TyG index thresholds and validate their prognostic value through longitudinal research.

**Trial Registration:**

PROSPERO: CRD42024547997

**Abbreviations**
2 h‐PG: 2‐h postprandial glucose
AUC: area under the curve
BC: breast cancer
BMI: body mass index
CAAs: cancer‐associated adipocytes
CI: confidence interval
EMT: epithelial‐to‐mesenchymal transition
HbA1c: glycated hemoglobin
HDL‐C: high‐density lipoprotein cholesterol
HPE: histopathological examination
HR: hazard ratio
HTN: hypertension
IGFs: insulin‐like growth factors
IL‐6: interleukin‐6
INSCOC: investigation on nutrition, screening, and outcome in Chinese patients
IR: insulin resistance
LDL‐C: low‐density lipoprotein cholesterol
MetS: metabolic syndrome
NA: not available
NOS: Newcastle‐Ottawa Scale
OR: odds ratio
PI3K/AKT: phosphoinositide 3‐kinase/protein kinase B
PRISMA: Preferred Reporting Items for Systematic Reviews and Meta‐Analyses
Ras/MAPK: rat sarcoma/mitogen‐activated protein kinase
RCS: restricted cubic splines
REML: restricted maximum likelihood
ROS: reactive oxygen species
SBP: systolic blood pressure
SHBG: sex hormone‐binding globulin
TC: total cholesterol
TG: triglycerides
TNF‐α: tumor necrosis factor‐alpha
TyG: triglyceride‐glucose
VEGF: vascular endothelial growth factor
WHR: waist‐to‐hip ratio
WMD: weighted mean difference

## 1 Introduction

Breast cancer (BC) is the most frequently diagnosed malignancy and the leading cause of cancer‐related deaths among women worldwide, with over 685 000 fatalities reported in 2020 alone. Projections suggest that by 2040, the global burden of BC will rise to an estimated 3 million new cases annually, underscoring the urgency of improving preventive and diagnostic measures [1, 2]. While early detection significantly enhances survival outcomes, with localized BC having a 5‐year survival rate of approximately 99%, identifying reliable markers for risk stratification remains a critical area of research [3, 4].

Metabolic syndrome (MetS), characterized by a constellation of interrelated conditions, including insulin resistance (IR), hyperglycemia, hypertension (HTN), and dyslipidemia, has emerged as a significant contributor to BC risk [5]. MetS provides a pro‐inflammatory and hyperinsulinemic state, creating a microenvironment conducive to tumor growth and progression [6, 7, 8]. Insulin and insulin‐like growth factors (IGFs) drive oncogenic pathways, such as the PI3K/AKT and Ras/MAPK cascades, which promote cellular proliferation and inhibit apoptosis [9, 10]. Moreover, low‐grade inflammation increases adipokine levels, which regulate the transcription of proto‐oncogenes and anti‐apoptotic pathways [11]. Given the growing prevalence of MetS globally, identifying accessible and cost‐effective markers to evaluate its impact on BC risk is of paramount importance.

The Triglyceride‐Glucose (TyG) index, calculated using the formula Ln [fasting triglycerides (mg/dL) × fasting glucose (mg/dL)/2], has gained attention as a surrogate marker for IR and MetS [12]. Its simplicity and strong correlation with MetS components, including HTN and central obesity, make it a potentially valuable tool in clinical practice [13, 14]. A recent meta‐analysis showed that higher TyG notably increases cancer risk [10]. However, studies have reported conflicting results regarding the association between BC and the TyG index. While two cohort studies found no significant association [15, 16], others have reported a higher risk of BC in individuals with an elevated TyG index [17, 18]. Moreover, the ability of the TyG index to differentiate between benign and malignant lesions has not been comprehensively evaluated.

This systematic review and meta‐analysis aim to bridge this knowledge gap by synthesizing available evidence on the relationship between the TyG index and BC risk. It investigates not only the association between elevated TyG index levels and BC incidence but also its utility in distinguishing malignant from benign breast lesions. By addressing these objectives, this study seeks to elucidate the clinical relevance of the TyG index as a cost‐effective and accessible marker for BC risk assessment and diagnostic refinement.

## 2 Methods

### 2.1 Protocol Registration

This study was conducted following PRISMA guidelines [19].

### 2.2 Inclusion and Exclusion Criteria

Studies were included if they met the following criteria: 1. *Population*: The study assessed patients with BC, with no restrictions on age, menopausal status, or pathological and immunohistological characteristics of the BC (e.g., receptor status such as ER, PR, or HER2); 2. *Intervention*: The study investigated the association between the TyG index as a marker of MetS and any aspect of BC, including its risk, progression, metastasis, or recurrence; 3. *Comparison*: The study included healthy individuals, patients with benign breast lesions, or pre‐specified internal control groups as comparators for patients with breast malignancy; 4. *Outcomes*.

#### 2.2.1 Primary Outcomes

1. The TyG index's predictive value in differentiating malignant and benign breast lesions is reported through diagnostic accuracy metrics such as sensitivity, specificity, area under the curve (AUC), and optimal cutoff values.
2. The association between the TyG index and BC risk, tumor characteristics, or clinical outcomes such as metastasis and recurrence.

#### 2.2.2 Secondary Outcomes

- The TyG index's predictive value in the early detection of undiagnosed BC within a general population highlights its potential role in screening or risk stratification.

Studies were excluded if they: (1) *Study type*: Were in vitro studies, animal studies, reviews, letters, recommendations, or case reports, gray literature. (2) *Insufficient data*: did not provide the data on the mean TyG index and SD in patients with breast malignancy and the control group or effect sizes (odds ratio [OR] and hazard ratio [HR]) and their 95% confidence interval (CI).

### 2.3 Search Strategy

A comprehensive search of online databases, including PubMed, Scopus, Web of Science, Embase, and Google Scholar, was conducted up to January 25, 2025. The search terms included combinations of the following keywords: “breast cancer,” “metabolic syndrome,” “Triglyceride‐Glucose index,” “TyG index,” and “insulin resistance.” Boolean operators (AND/OR) were used to combine the search terms. Studies were screened based on their titles and abstracts. The complete search strategy is detailed in the Supporting Information.

Two independent investigators (D.Z. and P.S.) screened the study in two stages: an initial review of titles and abstracts, followed by a full‐text review. Disagreements were resolved through consultation with a third reviewer (H.R.). No restrictions were applied regarding language or publication period. The included studies were imported into EndNote (version 21.2). Detailed information about the search strategy is provided in Table S1.

### 2.4 Data Extraction

A standardized data extraction form was developed. Two independent investigators (D.Z. and P.S.) extracted the following information from each study. D.Z. first extracted the information from all included studies, and P.S. rechecked the extracted information to ensure completeness and accuracy. Any disagreements were resolved by H.R.

Extracted information of each study: 1. *Study characteristics*: First author, study design, sample size, country, and year of publication; 2. *Population characteristics*: Gender, menopausal status, population source, mean age, presence of diabetes mellitus and HTN, lifestyle factors (including regular physical activity, alcohol consumption, and smoking status), TyG index and its standard deviation (SD) for BC patients and the control group, and family history of any malignancy; 3. *Outcome information*: Type of outcome, method of outcome assessment, and effect sizes related to BC, including HR and OR with 95% CI. 4. *Intervention and comparison information*: Mean and SD of the TyG index in patients with BC and the control group, the number of patients in each group, the timing of TyG index measurement, and factors adjusted for in the analysis.

### 2.5 Quality Assessment and Risk of Bias

The quality of each included study was evaluated utilizing the Newcastle‐Ottawa Scale (NOS), which assesses the methodological rigor of observational research. This scale assigns a maximum score of nine points based on three domains: participant selection, comparability of study groups, and assessment of outcomes and exposure. Specifically, four points are allocated for selection criteria, two points for comparability, and three points for how outcomes were measured and reported. Studies that obtained scores ranging from 7 to 9 were considered high quality, those with scores of 5 to 6 were classified as moderate quality, and studies receiving 4 or fewer points were regarded as low quality [20]. Only studies with an NOS score of 5 or higher were eligible for inclusion in the final analysis, ensuring that our review was based on adequately robust research.

### 2.6 Statistical Analysis

The weighted mean difference (WMD) was employed to compare the mean (SD) of the TyG index in patients with breast malignancy to that of the control group, provided that the TyG index was calculated using the same formula across all studies. To estimate the overall effect size, the natural logarithm (ln) of effect sizes (OR and HR) and their corresponding 95% CI were pooled to assess the risk of BC. The pooled effect size was reported as the exponentiated effect size along with its 95% CI. A random‐effects model was applied to account for both between‐study and within‐study variations. Heterogeneity among studies was evaluated using the *Q*‐statistic and the *I* ^2^ statistic. A *p*‐value of < 0.05 was considered indicative of significant heterogeneity [21]. To further investigate heterogeneity, a sensitivity analysis was conducted by sequentially omitting individual studies to evaluate their influence on the overall effect estimates. Additionally, the potential for publication bias was assessed visually through funnel plot symmetry and quantitatively using Egger's test.

To evaluate the dose–response relationship between the TyG index and BC risk, we employed a one‐stage approach utilizing restricted cubic splines (RCS) [22]. This method is well‐suited for capturing potential non‐linear associations between continuous exposures and disease risk. The analysis required known TyG index values along with corresponding risk estimates and their variance for at least two quantitative exposure categories. In cases where original studies did not explicitly report quantitative TyG values, we adopted a standardized approach for data extraction. If the mean or median TyG value was available for each category, we directly used these values. However, when only exposure ranges were reported, we estimated the category midpoint by calculating the arithmetic mean of the upper and lower boundaries. If a category was open‐ended (e.g., the highest or lowest category lacked a defined boundary), we assumed that the interval width was equivalent to the adjacent category to maintain consistency and comparability across studies [23].

A two‐stage random‐effects meta‐analysis was performed using the restricted maximum likelihood (REML) estimation method. Initially, a linear dose–response meta‐analysis was performed, assuming a constant increase in BC risk per unit increase in TyG. To evaluate potential non‐linearity, an RCS model with three knots (placed at predetermined percentiles of the TyG distribution) was applied. The non‐linearity was formally tested using a Wald test [24]. The meta‐analysis was performed using STATA (version 17.0) and R (version 4.4.2), with statistical significance defined as a *p*‐value of < 0.05.

## 3 Results

### 3.1 Search Results

**FIGURE 1:**
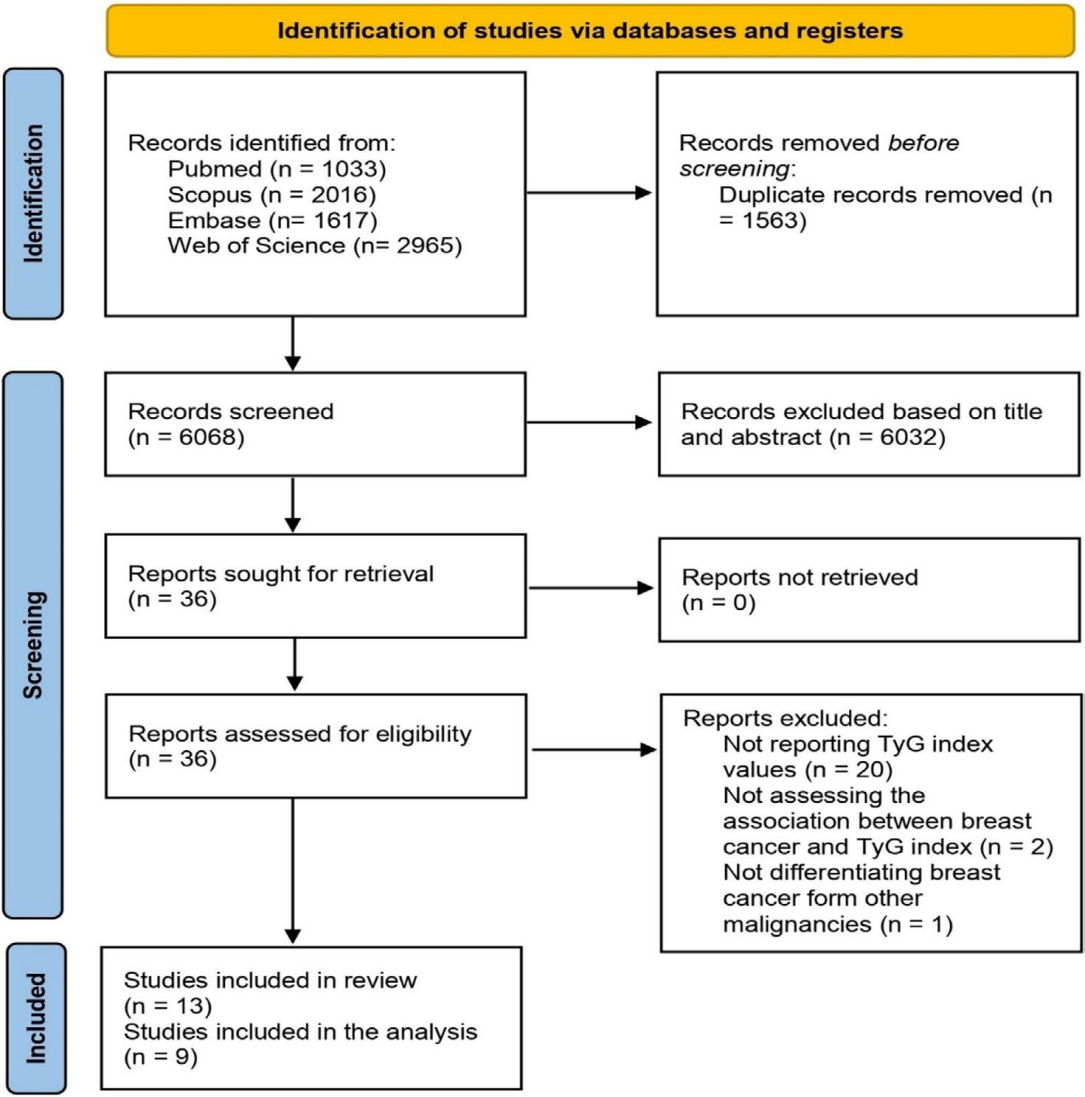
PRISMA flow diagram of study selection. This diagram illustrates the systematic process of identifying, screening, and including studies in the meta‐analysis, detailing the number of records at each stage and reasons for exclusion.

Initially, 7631 studies were identified through a comprehensive search. After the removal of 1563 duplicates, 6068 records remained for title and abstract screening. During the first screening stage, 6032 studies were excluded. Subsequently, 36 full‐text articles were assessed, of which 23 were excluded due to insufficient data linking BC with the TyG index. Following a detailed evaluation, 13 studies were included in this review. The selection process is depicted in Figure 1. The reasons for exclusion have been reported in Appendix S1.

### 3.2 Baseline Characteristics of Included Studies

This systematic review included retrospective (*n* = 2) [25, 26], case–control (*n* = 1) [17], cohort (*n* = 3) [15, 16, 18], and cross‐sectional (*n* = 7) [27, 28, 29, 30, 31, 32, 33] studies. The total number of participants ranged from 88 to 510 741 individuals. All included studies assessed both premenopausal and postmenopausal individuals. Additionally, two studies evaluated males with BC. The studies were conducted in Brazil (*n* = 1), Asia (*n* = 7), Europe (*n* = 4), and the United States (*n* = 1). Most of the studies did not report information regarding family history of cancer, lifestyle factors, or underlying chronic diseases. Three cohort studies assessed the TyG index before diagnosing BC, while others reported the post‐diagnosis TyG index. Table 1 presents detailed characteristics of the included studies.

**TABLE 1:** Baseline characteristics of the included studies.

First author (year)	Design	Sample size	Menopausal status	Male/female	Population source	Country	Study perioud	Mean follow‐up time	Mean age (both group)	Diabates (%)	HTN (%)	Family history of malignancy (%)	Regular physical activity	Current or previous smoking (%)	Drinking (%)	The TyG index measurement time	Outcome	Outcome assessment	Adjusted cofounders
Karadag (2023) [25]	Retrospective	120	Both	0/120	Dr. Abdurrahman Yurtaslan Hospital, Ankara/Turkey	Turkey	2000–2021	NA	48	0	0	NA	NA	10.8	NA	Post‐diagnosis	Prognostic value of TyG index to predict time to brain metastasis at HER2 positive breast cancer	NA	NA
Önder (2024) [26]	Retrospective	333	Both	0/333	Dr. Abdurrahman Yurtaslan Hospital, Ankara/Turkey	Turkey	2017–2023	NA	56	NA	NA	NA	NA	19.8	NA	Post‐diagnosis	Prognostic value of TyG index to predict time to metastasis at HER2 negative breast cancer	HPE	NA
Alkurt (2022) [27]	Cross‐sectional	510	Both	13/499	Computer records	Turkey	2018–2021	NA	51	NA	NA	NA	NA	NA	NA	Post‐diagnosis	Predictive value of TyG to differentiate malignant and benign lesions	HPE	NA
da Silva (2022) [28]	Cross‐sectional	88	Both	0/88	General Hospital of School of Medicine of Ribeirão Preto, São Paulo, Brazil	Brazil	NA	NA	45	NA	NA	NA	NA	NA	NA	Post‐diagnosis	Predictive value of TyG to differentiate malignant and benign lesions	HPE	Age, gender, and BMI
Rachman (2023) [29]	Cross‐sectional	150	Both	0/150	Cipto Mangunkusumo and MRCCC Hospital, Jakarta, Indonesia	Indonesia	NA	NA	NA	NA	NA	NA	NA	NA	NA	Post‐diagnosis	Prognostic value of TyG index to predict metastasis breast cancer	HPE	NA
Rajakumar (2024) [30]	Cross‐sectional	200	Both	0/200	Government Medical College, Omandurar, Chennai	India	2023	NA	28.8, 52.8	0	NA	NA	NA	NA	NA	Post‐diagnosis	Distinguishing benign and malignant tumors among patients with complaints of breast mass	Cytological and HPE analyses of biopsy specimens	NA
Shi (2022) [31]	Cross‐sectional	11 466	Both	0/11 466	National Health and Nutrition Examination Survey (NHANSE 1999–2018)	USA	1999–2018	NA	20–85	10.8	34.3	NA	NA	NA	NA	Post‐diagnosis	Risk of breast cancer	Database	Age, Race, BMI, LDL‐C, HDL‐C, Marital status, Education, Age at menarche, Age at menopause, Diabetes, Hypertension, and Breastfeeding history
Wu (2024) [32]	Cross‐sectional	142 184	Both	0/142 184	The REACTION study	China	NA	NA	56.37	NA	NA	6.3	8.75	1.3	2.2	Post‐diagnosis	Risk of breast cancer	Database	Age, BMI, smoking status, drinking status, physical activity, family history of breast cancer, healthy diet, 2 h‐PG, HbA1c and HDL‐C, age at menarche, menopausal status, and number of childbirths and breastfeeding
Zhang (2024) [33]	Cross‐sectional	2111	Both	0/2111	Department of Surgery, Women's Hospital, Zhejiang University School of Medicine	China	2020–2021	NA	42.17	NA	NA	15.8	NA	1.78	2.74	Post‐diagnosis	Risk of breast cancer	Database	Age, BMI, smoking status, drinking status, hypertension, family history of malignancy, age at menarche, and hormonal contraception
Fritz (2020) [15]	Cohort	510 741	Both	257/252 503	Six European cohort studies	Six Europran countries	2012–2014	17.2	43.1	NA	NA	NA	NA	52.6	NA	Pre‐diagnosis	Risk of obesity‐related cancers	HPE	Age, sex, smoking status, fasting status, cohort and decade of birth, and BMI
Li (2024) [16]	Cohort	27 604	Both	0/27 604	Kailuan Group	China	2006–2010	12.9	47.53	NA	NA	NA	1.8	6.3	13.1	Pre‐diagnosis	Risk of breast cancer	HPE	Age, SBP, WHR, TG, TC, frequency of physical exercise, smoking, alcohol consumption, and salt intake
Liu (2024) [18]	Cohort	571	Both	0/571	INSCOC cohort in China	China	NA	NA	52	6	16.5	16.5	5.8	NA	3	Pre‐diagnosis	Risk of all‐cause mortality	NA	age, tumor stage, surgery, chemotherapy, radiotherapy, albumin
Panigoro (2021) [17]	Case–control	424	Both	0/424	Six public referral hospitals in Indonesia	Indonesia	NA	NA	47	NA	NA	16.5	NA	38.9	7.5	Post‐diagnosis	Risk of breast cancer	HPE	NA

Abbreviations: 2 h‐PG, 2‐h postprandial glucose; BC, breast cancer; BMI, body mass index; HbA1c, glycated hemoglobin; HDL‐C, high‐density lipoprotein cholesterol; HPE, histopathological examination; HTN, hypertension; INSCOC, investigation on nutrition, screening, and outcome in Chinese patients; LDL‐C, low‐density lipoprotein cholesterol; NA, not available; SBP, systolic blood pressure; TC, total cholesterol; TG, triglycerides; TyG, triglyceride‐glucose; WHR, waist‐to‐hip ratio.

### 3.3 Findings From Meta‐Analysis

#### 3.3.1 TyG Index and Risk of Breast Cancer

**FIGURE 2:**
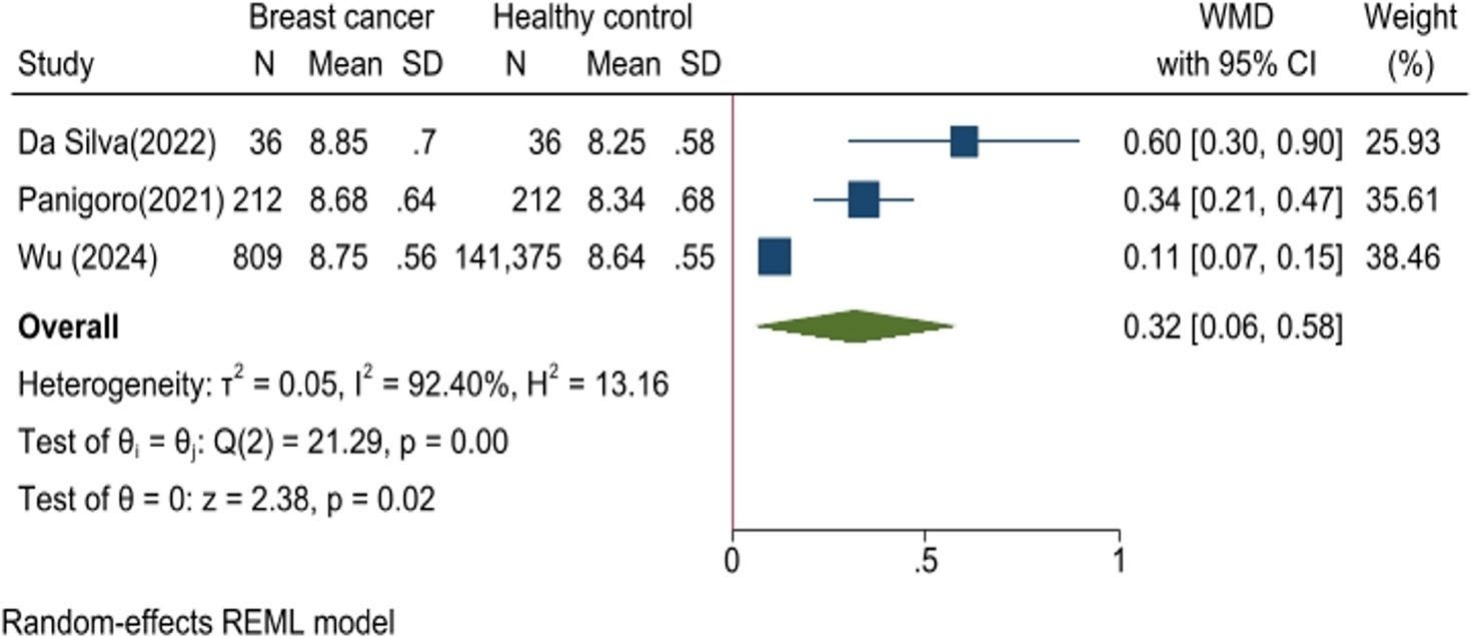
Forest plot comparing mean TyG index levels between breast cancer patients and healthy controls.

Three studies reported TyG values in patients diagnosed with BC compared to a healthy control group. The analysis demonstrated significantly higher levels of the TyG index in patients with BC compared to controls (WMD: 0.32, 95% CI: 0.06–0.58, *p* = 0.02, *I* ^2^: 92.40%, Figure 2). However, the sensitivity analysis indicated non‐robust findings (Figure S1). Additionally, the funnel plot and Egger's test suggested potential publication bias among the studies (*p* < 0.001) (Figure S2).

**FIGURE 3:**
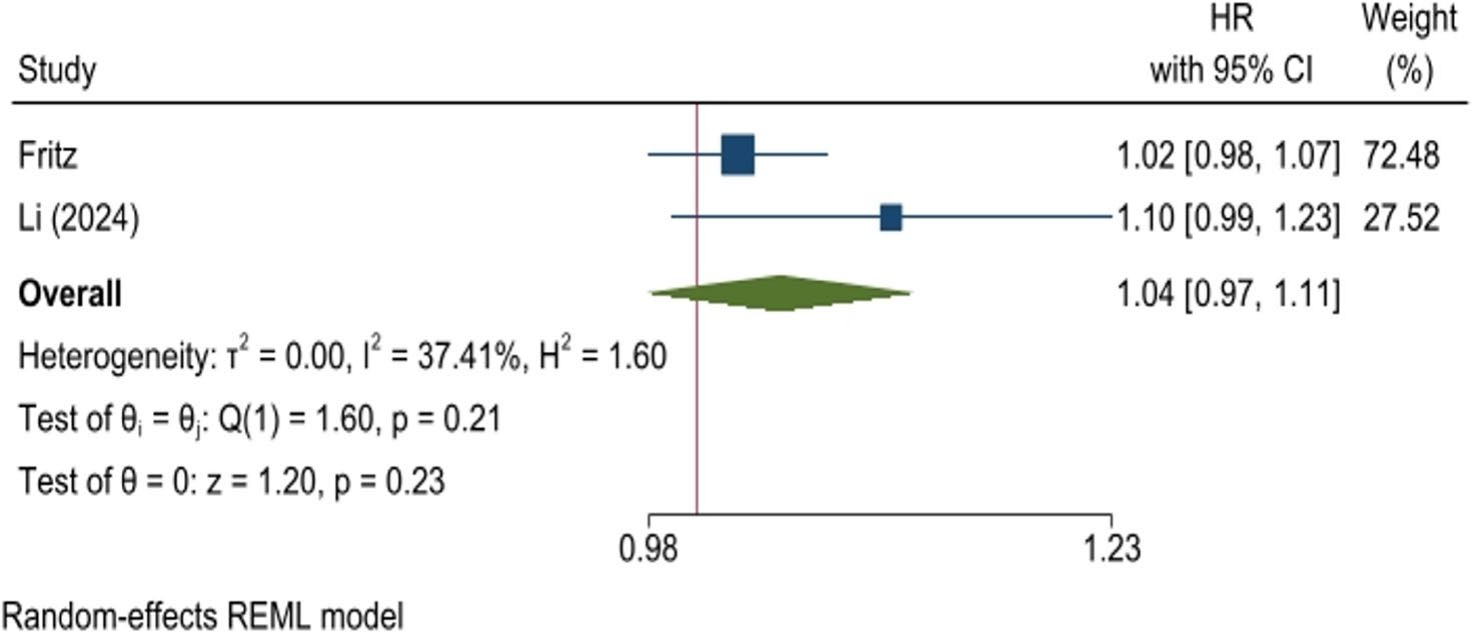
Hazard ratio (HR) for breast cancer risk associated with the TyG index in cohort studies. A pooled analysis of prospective cohort studies reveals no statistically significant association between the elevated TyG index and breast cancer risk.

**FIGURE 4:**
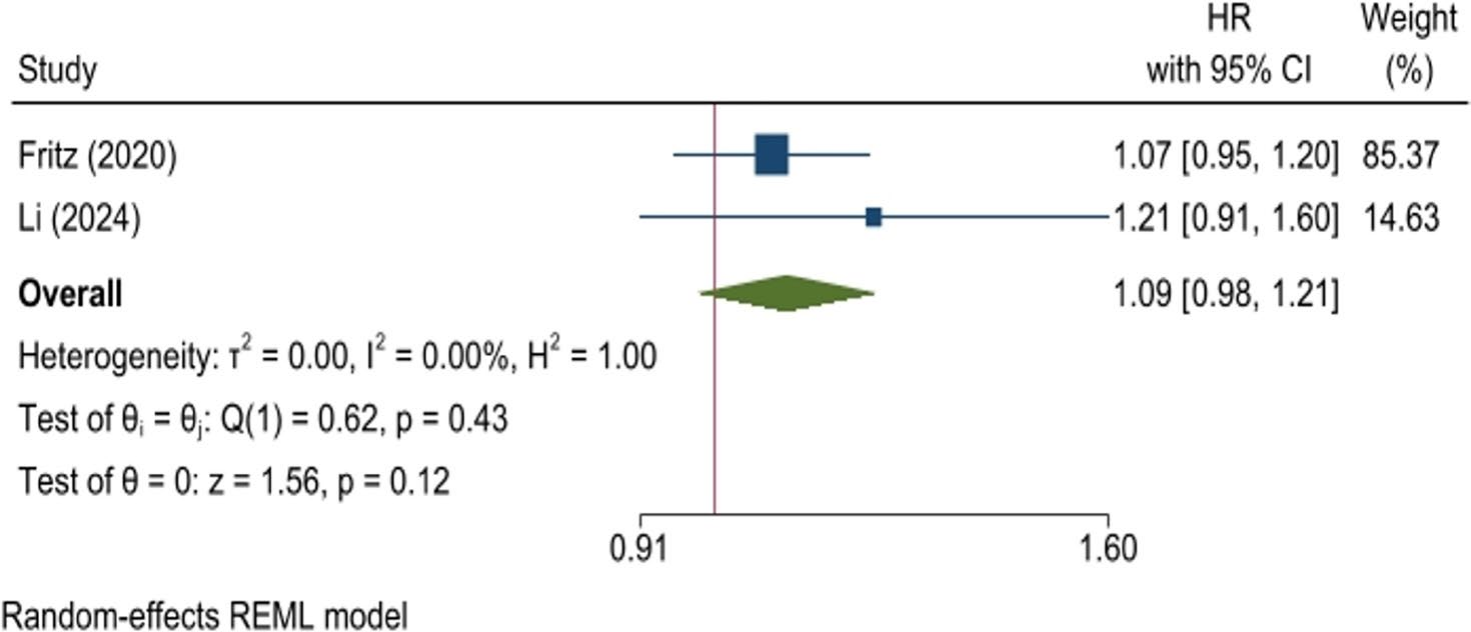
Risk of breast cancer in individuals with the highest TyG index compared to the lowest category. The meta‐analysis of cohort studies suggests no significant increase in breast cancer risk for individuals in the highest TyG index group.

A pooled analysis of cohort studies revealed no significant relationship between the risk of BC and an increasing TyG index (HR: 1.04, 95% CI: 0.97–1.11, *p* = 0.23, *I* ^2^: 37.41%, Figure 3). The risk of BC was also analyzed in patient groups with the highest TyG index compared to the lowest index group, used as the reference. Analysis showed no meaningful finding (HR: 1.09, 95% CI: 0.98–1.21, *p* = 0.12, Figure 4). Both analyses remained stable when excluding studies in the sensitivity analysis (Figures S3 and S5). Furthermore, the funnel plot indicated no publication bias (Figures S4 and S6).

**FIGURE 5:**
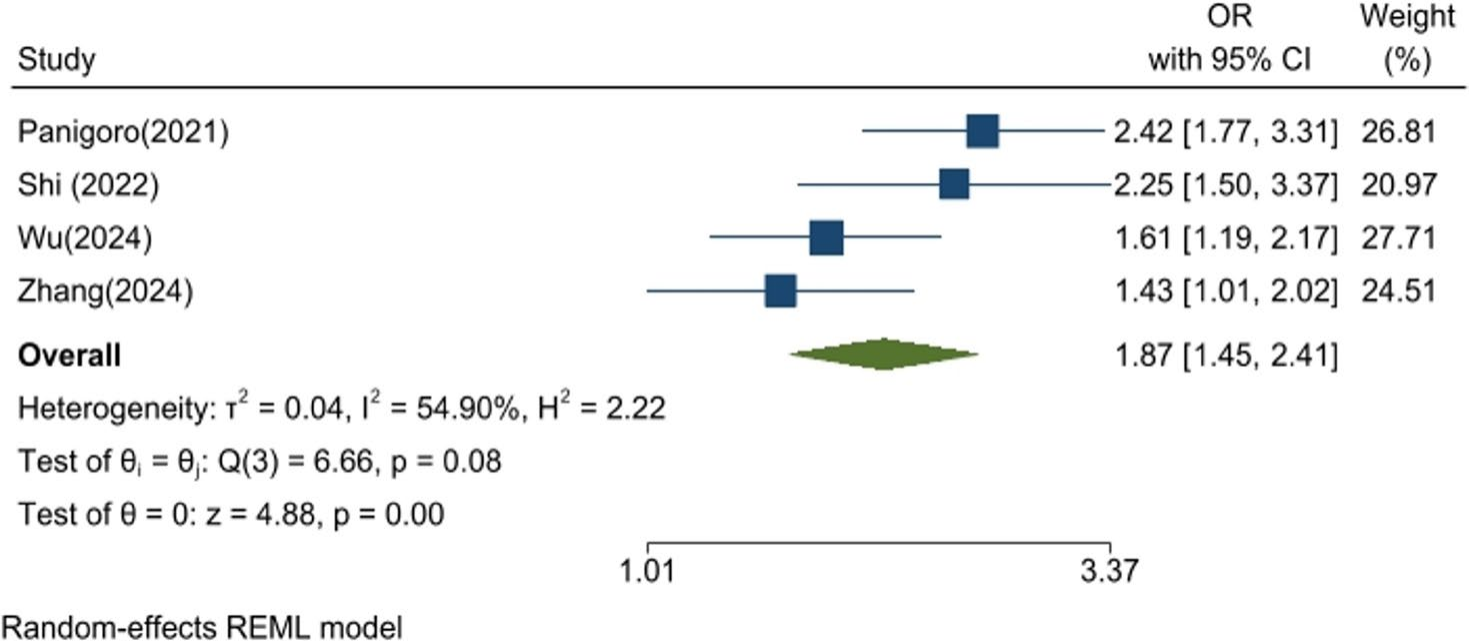
Forest plot showing the association between TyG index and breast cancer risk in case–control and cross‐sectional studies. The results indicate a significantly higher breast cancer risk in individuals with an elevated TyG index.

**FIGURE 6:**
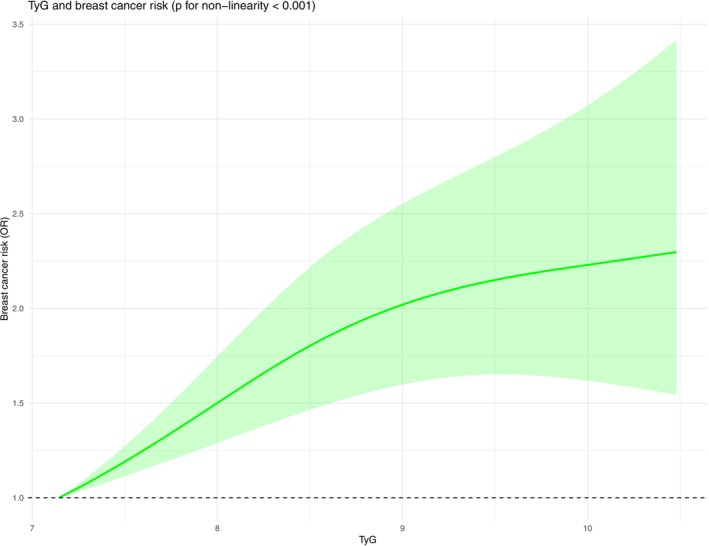
Dose–response relationship between the TyG index and breast cancer risk. The restricted cubic spline model suggests a non‐linear association, indicating that the risk of breast cancer increases disproportionately with higher TyG index levels.

However, the analysis of case–control and cross‐sectional studies demonstrated a significantly higher risk of BC in patients with the highest TyG index (OR: 1.87, 95% CI: 1.45–2.41, *p* < 0.01, *I* ^2^: 54.90%, Figure 5) than in the lowest group. The sensitivity analysis confirmed the robustness of the finding (Figure S7). Furthermore, the funnel plot and Egger's test suggested no publication bias (Figure S8) (*p* = 0.742). The linear dose–response meta‐analysis demonstrated a statistically significant positive association between TyG and breast cancer risk (*p* < 0.001). The restricted cubic spline model suggested a non‐linear relationship (*p* < 0.001). The Wald test confirmed that the non‐linear model provided a significantly better fit than the linear model (*p* = 0.015), supporting the presence of a non‐linear dose–response relationship (Figure 6).

#### 3.3.2 Differentiating Benign and Malignant Lesions

**FIGURE 7:**
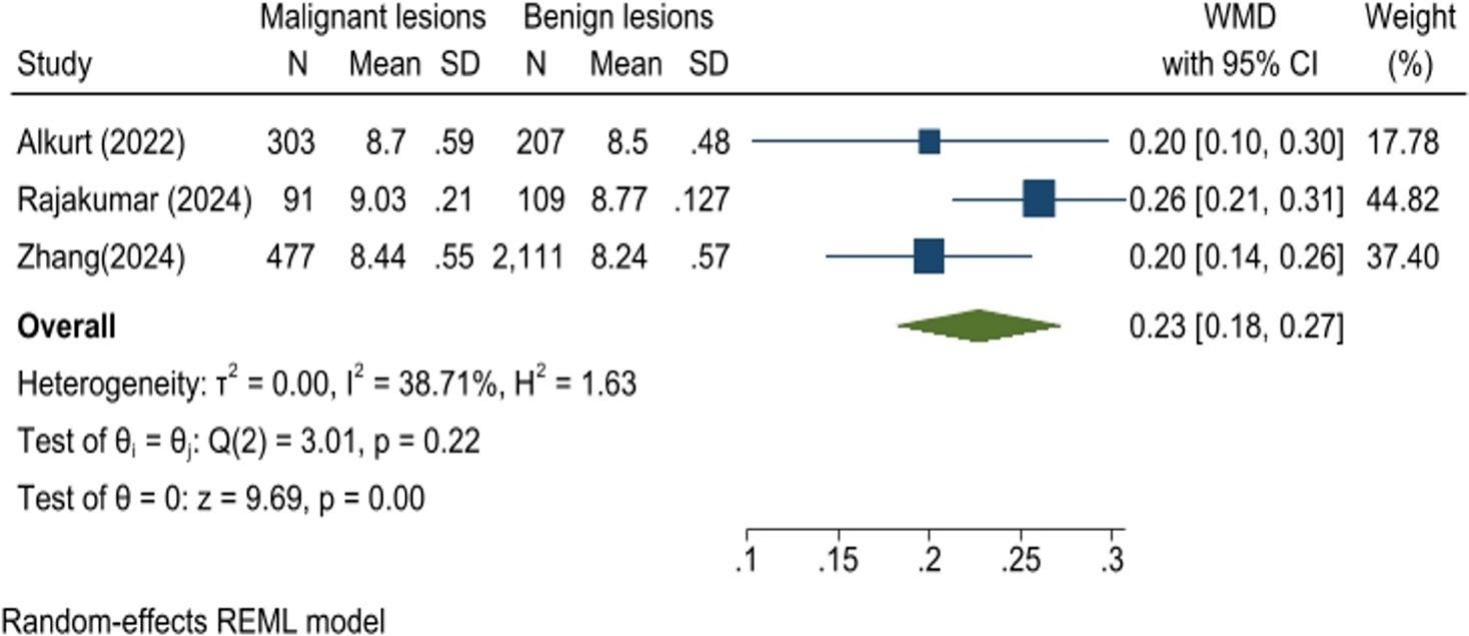
Comparison of TyG index levels in patients with malignant versus benign breast lesions.

**FIGURE 8:**
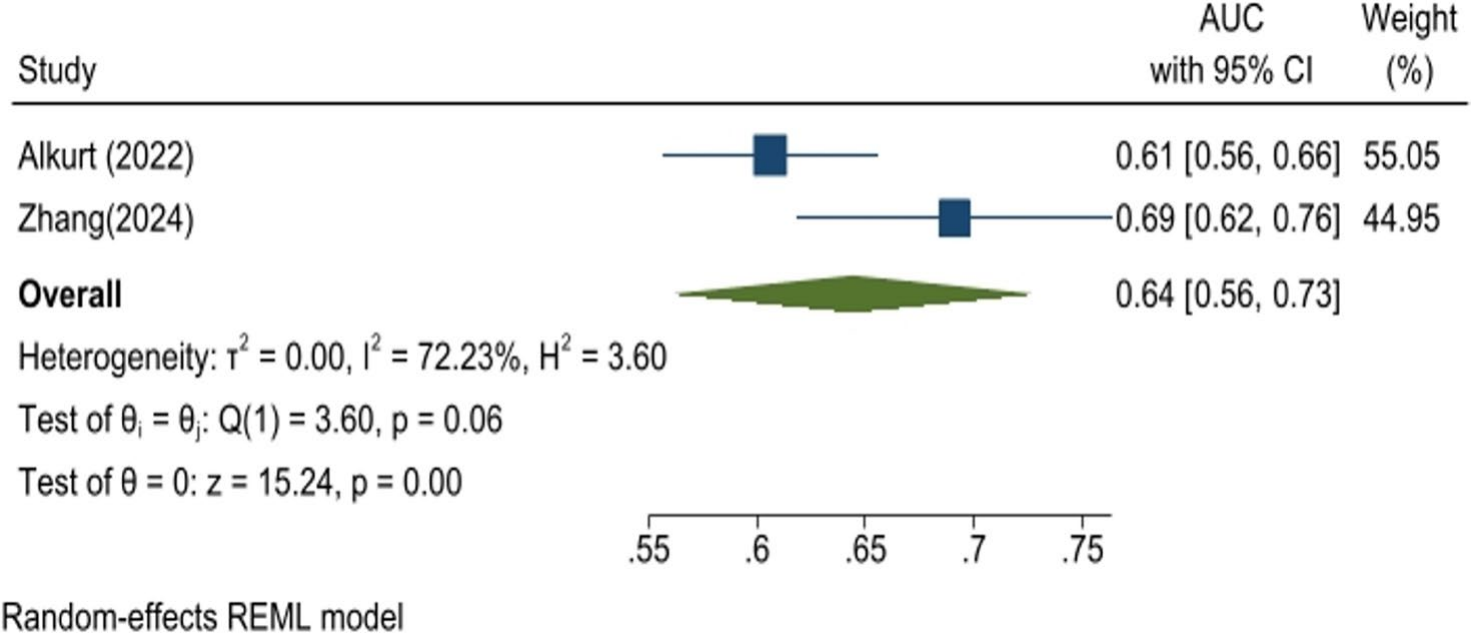
Forest plot showing the overall area under the curve (AUC) and 95% CI for the predictive value of TyG to differentiate benign and malignant lesions.

An analysis of three studies demonstrated a significantly higher TyG index in patients with malignant breast lesions compared to those with benign lesions (WMD: 0.23, 95% CI: 0.18–0.27, *p* < 0.01; Figure 7). A sensitivity analysis confirmed the robustness of these findings (Figure S9). Furthermore, Egger's test and the funnel plot revealed no evidence of publication bias among the studies (*p* = 0.435; Figure S10). The analysis also highlighted the strong predictive value of the TyG index for differentiating between malignant and benign breast lesions (AUC: 0.64, 95% CI: 0.56–0.73, *p* < 0.01; Figure 8). Rajakumar et al. reported an even stronger predictive value for the TyG index, with an AUC of 0.835, a sensitivity of 65.93%, and specificity of 93.58% at a cut‐off value of 8.95.

### 3.4 Findings From the Systematic Review

#### 3.4.1 TyG Index as a Prognostic Factor

The limited number and heterogeneity of the included studies precluded the performance of a meta‐analysis on the role of the TyG index in predicting metastasis and survival outcomes in patients with BC. The study by Liu et al. demonstrated a significantly higher risk of all‐cause mortality in patients with a TyG index greater than 4.62 compared to the reference group (HR: 1.64, 95% CI: 1.13–2.40, *p* = 0.010) [18]. Furthermore, this study reported a 19% increased risk of all‐cause mortality for every one‐unit increase in the TyG index (HR: 1.19, 95% CI: 1.01–1.41, *p* = 0.043) [18]. Consistently, another study found that overall survival and progression‐free survival were significantly lower in BC patients with a TyG index less than 8.43 (*p* = 0.022 and *p* = 0.030, respectively) [26].

However, the role of the TyG index as a potential marker for metastasis has shown varying results across studies. For instance, one study reported that patients with high TyG index values experienced a mean time to brain metastasis of 15 months, compared to 35 months for those with low TyG index values (*p* < 0.001) [25]. Conversely, Rachman et al. observed no significant difference in TyG index values between patients with metastasis and those without metastasis (*p* = 0.08) [30].

## 4 Discussion

This meta‐analysis highlights the complex relationship between the TyG index and BC. Case–control and cross‐sectional studies revealed a significant nonlinear association between elevated TyG index levels and BC risk, whereas cohort studies did not yield statistically significant findings. Additionally, the analysis of comparing the TyG index in patients with BC and controls was affected by substantial publication bias and limited sensitivity, necessitating cautious interpretation of the comparing TyG values between BC patients and controls. In contrast, the TyG index demonstrated a consistent and reliable predictive value in differentiating malignant from benign breast lesions, highlighting its potential as a diagnostic tool.

The findings of this meta‐analysis align with previous cohort studies, which were supported by long follow‐up periods and large sample sizes. These studies showed no meaningful relationship between BC risk and the TyG index measured before diagnosis [15, 16]. In contrast, other observational studies assessed the TyG index after BC diagnosis and reported a significantly higher risk of BC [17, 31]. A similar finding was also reported by the previous meta‐analysis, which reported a higher risk of overall cancer in patients with a higher TyG index [10]. This suggests that the TyG index may function more as a metabolic marker altered by cancer progression rather than as a predictor of cancer risk. Moreover, consistent with previous publications, the analysis also identified significantly higher TyG index levels in malignant breast lesions compared to benign ones, indicating that insulin resistance may contribute to BC progression.

The TyG index, a well‐established surrogate marker of IR and MetS, contributes to oncogenesis through multiple pathways. Chronic hyperinsulinemia downregulates sex hormone‐binding globulin (SHBG), leading to increased bioavailability of estrogen, a well‐known driver of BC, particularly in hormone receptor‐positive subtypes [34]. Additionally, IR and hyperglycemia enhance the Warburg effect, wherein cancer cells preferentially utilize glucose for rapid energy production, further fueling tumor growth [35]. MetS also promotes a chronic inflammatory state characterized by elevated pro‐inflammatory cytokines such as interleukin‐6 (IL‐6), tumor necrosis factor‐alpha (TNF‐α), and leptin, which enhance tumor progression by activating cancer‐associated adipocytes (CAAs) and modulating the tumor microenvironment [36, 37]. Concurrently, increased oxidative stress associated with MetS leads to DNA damage, genomic instability, and epigenetic alterations that drive carcinogenesis [38]. Thus, the TyG index serves as an accessible, indirect measure of these oncogenic metabolic and inflammatory processes.

The analysis revealed that the pre‐diagnostic TyG index was not significantly associated with BC risk. Several mechanisms may explain this finding. In the early stages of IR, the pancreas compensates by increasing insulin secretion, maintaining relatively stable glucose levels [39]. This suggests that while hyperinsulinemia may trigger tumorigenic pathways, glucose and triglyceride levels might not be significantly elevated [40]. Moreover, early tumorigenesis is driven by multiple local and systemic factors, reducing the detectable impact of the TyG index before diagnosis [41]. On the other hand, tumor cells exhibit heightened glucose and lipid uptake, driven by the Warburg effect and dysregulated lipid metabolism [42]. Additionally, cancer‐related inflammation, characterized by elevated cytokines such as TNF‐α and IL‐6, further disrupts insulin signaling, exacerbating metabolic dysfunction [43, 44]. Collectively, these mechanisms create a metabolic milieu in which the TyG index serves as a significant marker of BC progression and prognosis.

The role of the TyG index in predicting metastasis and survival outcomes remains inconclusive, with studies yielding conflicting results. For instance, one study reported a significantly shorter mean time to brain metastasis in BC patients with higher TyG index values [25], while another found no significant differences between metastatic and non‐metastatic groups [29]. Elevated TyG index levels may contribute to metastasis through multiple mechanisms. IR and hyperglycemia promote epithelial‐to‐mesenchymal transition (EMT), a critical step in metastatic progression [45]. Insulin and IGF‐1 also enhance angiogenesis via VEGF signaling, facilitating tumor vascularization and dissemination [46, 47]. Furthermore, hyperglycemia‐induced glycation products and reactive oxygen species (ROS) create a pre‐metastatic niche by modulating the extracellular matrix and enabling tumor cell migration [48]. However, the lack of consistent findings suggests that these effects may vary across BC subtypes and stages, necessitating future research using prospective designs and standardized methodologies.

The TyG index showed consistent utility in differentiating malignant from benign breast lesions. Its diagnostic value is particularly promising in limited settings, where access to advanced imaging technologies may be restricted. Malignant lesions are often characterized by increased metabolic activity, including heightened glucose uptake and lipid turnover, reflected in elevated TyG index values [49]. Cancer cells rely heavily on glycolysis and lipogenesis to sustain rapid growth, processes indirectly captured by the TyG index [50]. These metabolic differences likely account for its ability to distinguish between malignant and benign lesions. Additionally, the pro‐inflammatory milieu associated with malignancy further elevates the TyG index. To fully harness its diagnostic potential, future studies should focus on standardizing TyG index thresholds, validating its use across diverse populations, and integrating it into existing diagnostic workflows alongside imaging and molecular profiling.

## 5 Limitations and Future Directions

This study has several limitations that warrant consideration. First, significant publication bias was detected, which may have skewed the findings regarding BC risk. Second, variability in study designs, populations, and methodologies complicates result interpretation. Third, the absence of consistent prospective data limits the ability to establish causal relationships between the TyG index and BC risk, metastasis, or survival. Finally, key confounders such as lifestyle factors, socioeconomic status, and other metabolic markers were not uniformly adjusted for in the included studies.

To address these limitations and advance research on the TyG index in BC, the following areas are recommended for future studies:

1. Mediation Analyses: Evaluate whether the TyG index independently predicts BC risk or reflects broader metabolic dysfunction by adjusting for key mediators like insulin levels and inflammatory markers.
2. Standardization: Develop global and population‐specific thresholds for the TyG index to enhance clinical applicability
3. Longitudinal Studies: Conduct prospective cohort studies to explore dynamic changes in the TyG index and their association with BC risk, progression, metastasis, and survival.
4. Integration with Diagnostics: Assess the utility of the TyG index as part of a multimodal approach, combining it with imaging, molecular biomarkers, and other metabolic indicators.

## 6 Conclusion

This systematic review and meta‐analysis highlight the potential clinical relevance of the TyG index in BC assessment. While case–control and cross‐sectional studies suggest a strong association between an elevated TyG index and increased BC risk, cohort studies did not confirm this relationship, indicating that the TyG index may be more reflective of metabolic alterations associated with cancer progression rather than a direct predictor of BC onset. Importantly, the TyG index demonstrated significant utility in distinguishing between benign and malignant breast lesions, suggesting its potential as a cost‐effective and easily accessible biomarker in clinical settings. Given the increasing global burden of BC and the need for improved risk stratification, the TyG index could serve as an adjunct to existing diagnostic tools, particularly in resource‐limited settings. Future research should focus on establishing standardized thresholds, integrating the TyG index into clinical workflows, and assessing its prognostic value for BC progression and survival outcomes.

## Author Contributions

All authors had full access to the data in the study and take responsibility for the integrity of the data and the accuracy of the data analysis. Conceptualization: H.R., B.S.A. and D.Z.; Methodology: D.Z.; Investigation: H.R., B.S.A., P.S. and D.Z.; Formal analysis: D.Z.; Resources: P.S.; Writing – original draft: D.Z. and P.S.; Writing – review and editing: H.R., B.S.A., P.S. and D.Z.; Visualization: H.R. and B.S.A.; Supervision: H.R. and B.S.A.; Funding acquisition: None.

## Conflicts of Interest

The authors declare no conflicts of interest.

## Supporting information


Appendix S1

Appendix S2

Appendix S3

Figures S1–S10

Table S1

## Data Availability

The datasets generated and analyzed during the current study are available from the corresponding author on reasonable request.
