# Supplementary material for: Association Between Triglyceride‐Glucose Index and Breast Cancer: A Systematic Review and Meta‐Analysis
Source: Cancer Rep (Hoboken). 2025 Apr 7;8(4):e70194. doi: 10.1002/cnr2.70194 (PMC11976027; doi:10.1002/cnr2.70194)
Supplement: Supplementary file 1 — Appendix S1 [file CNR2-8-e70194-s001.docx]

| **First author (year)** | **Reasons for exclusion** |
| --- | --- |
| Aykol (2017) [1] | This study didn’t provide the TyG index values in individuals |
| Bell (2014) [2] | This study didn’t provide the TyG index values in individuals |
| Berstein (2012) [3] | This study didn’t provide the TyG index values in individuals |
| Burt (2021) [4] | This study didn’t provide the TyG index values in individuals |
| Buttors (2012) [5] | This study didn’t provide the TyG index values in individuals |
| Dieli-Conwright (2016) [6] | This study didn’t provide the TyG index values in individuals |
| Gathirua-Mwangi (2018) [7] | This study didn’t provide the TyG index values in individuals |
| Guinan (2013) [8] | This study didn’t provide the TyG index values in individuals |
| Healy (2010) [9] | This study didn’t provide the TyG index values in individuals |
| Jung (2022) [10] | This study didn’t assess the relationship between breast malignancy and TyG index |
| Kim (2019) [11] | This study didn’t provide the TyG index values in individuals |
| Michelsen (2009) [12] | This study didn’t provide the TyG index values in individuals |
| Mohammadbeigy (2023) [13] | This study didn’t provide the TyG index values in individuals |
| Motoki (2021) [14] | This study didn’t provide the TyG index values in individuals |
| Ortiz-Mendoza (2014) [15] | This study didn’t provide the TyG index values in individuals |
| Ruan (2024) [16] | This study reported the mean and standard deviation (SD) of TyG index in patients with various malignancies. However, data specific to patients with breast cancer were not provided separately. |
| Rezzonico (2007) [17] | This study didn’t provide the TyG index values in individuals |
| Shi (2023)[18] | This study assessed the association between cardiac remodeling and TyG index in HER2-positive breast cancer patients. However, they did not include any control group |
| Terra Branco (2019) [19] | This study didn’t provide the TyG index values in individuals |
| Thomson (2009) [20] | This study didn’t provide the TyG index values in individuals |
| Wung (2015) [21] | This study didn’t provide the TyG index values in individuals |
| Yoon (2015) [22] | This study didn’t provide the TyG index values in individuals |
| Zhou (2022) [23] | This study didn’t provide the TyG index values in individuals |

[1] Akyol M, Alacacioglu A, Demir L, Kucukzeybek Y, Yildiz Y, Gumus Z, et al. The alterations of serum FGF-21 levels, metabolic and body composition in early breast cancer patients receiving adjuvant endocrine therapy. Cancer Biomark. 2017;18:441-9.

[2] Bell KE, Di Sebastiano KM, Vance V, Hanning R, Mitchell A, Quadrilatero J, et al. A comprehensive metabolic evaluation reveals impaired glucose metabolism and dyslipidemia in breast cancer patients early in the disease trajectory. Clin Nutr. 2014;33:550-7.

[3] Berstein LM, Boyarkina MP, Vasilyev DA, Poroshina TE, Kovalenko IG, Imyanitov EN, Semiglazov VF. Endocrine metabolic disorders in patients with breast cancer, carriers of BRCA1 gene mutations. Bull Exp Biol Med. 2012;152:610-2.

[4] Burt MG, Mangelsdorf BL, Drake SM, Swan M, Padman S, Vatandoust S, Koczwara B. Insulin sensitivity, cardiovascular function and bone health in women with early stage breast cancer before and after cancer treatment. Intern Med J. 2022;52:1917-24.

[5] Buttros Dde A, Nahas EA, Vespoli Hde L, Uemura G, de Almeida Bda R, Nahas-Neto J. Risk of metabolic syndrome in postmenopausal breast cancer survivors. Menopause. 2013;20:448-54.

[6] Dieli-Conwright CM, Wong L, Waliany S, Bernstein L, Salehian B, Mortimer JE. An observational study to examine changes in metabolic syndrome components in patients with breast cancer receiving neoadjuvant or adjuvant chemotherapy. Cancer. 2016;122:2646-53.

[7] Gathirua-Mwangi WG, Song Y, Monahan PO, Champion VL, Zollinger TW. Associations of metabolic syndrome and C-reactive protein with mortality from total cancer, obesity-linked cancers and breast cancer among women in NHANES III. Int J Cancer. 2018;143:535-42.

[8] Guinan EM, Connolly EM, Kennedy MJ, Hussey J. The presentation of metabolic dysfunction and the relationship with energy output in breast cancer survivors: a cross-sectional study. Nutr J. 2013;12:99.

[9] Healy LA, Ryan AM, Carroll P, Ennis D, Crowley V, Boyle T, et al. Metabolic syndrome, central obesity and insulin resistance are associated with adverse pathological features in postmenopausal breast cancer. Clin Oncol (R Coll Radiol). 2010;22:281-8.

[10] Jung MH, Yi SW, An SJ, Yi JJ, Ihm SH, Han S, et al. Associations between the triglyceride-glucose index and cardiovascular disease in over 150,000 cancer survivors: a population-based cohort study. Cardiovasc Diabetol. 2022;21:52.

[11] Kim HJ, Kim HS, Kim HR, Yoo YS, Song BJ. Characterization of Metabolic Syndrome Risk Factors and Health-Related Behaviors in Korean Patients With Breast Cancer by Abdominal Obesity Status. J Nurs Res. 2020;28:e74.

[12] Michelsen TM, Pripp AH, Tonstad S, Tropé CG, Dørum A. Metabolic syndrome after risk-reducing salpingo-oophorectomy in women at high risk for hereditary breast ovarian cancer: a controlled observational study. Eur J Cancer. 2009;45:82-9.

[13] Mohammadbeigy I, Khalilian MS, Najafizadeh N, Moazam E, Hemati S, Zeinalian M. The role of serum lipid profile, fasting blood sugar, and body mass index on recurrence and metastasis in patients with estrogen receptor-positive breast cancer: A case-control study. J Res Med Sci. 2023;28:83.

[14] Motoki AH, Buttros DAB, Gaspar AL, Almeida-Filho BS, Carvalho-Pessoa E, Vespoli HDL, et al. Association Between Metabolic Syndrome and Immunohistochemical Profile at Breast Cancer Diagnosis in Postmenopausal Women. Clin Breast Cancer. 2022;22:e253-e61.

[15] Ortiz-Mendoza CM, de-la-Fuente-Vera TA, Pérez-Chávez E. Metabolic syndrome in Mexican women survivors of breast cancer: a pilot study at a general hospital. Med Arch. 2014;68:19-21.

[16] Ruan GT, Xie HL, Zhang HY, Liu CA, Ge YZ, Zhang Q, et al. A Novel Inflammation and Insulin Resistance Related Indicator to Predict the Survival of Patients With Cancer. Front Endocrinol (Lausanne). 2022;13:905266.

[17] Rezzonico JN, Sayegh F, Rezzonico M, Pusiol E, Gago FE, Masia Francés E, et al. Insulin resistance and familial history of breast cancer. Endocrinología y Nutrición. 2007;54:288-93.

[18] Shi Y, Qiu Z, Yu J, Li Z, Hua S, Chen Y, et al. Association between insulin resistance and cardiac remodeling in HER2-positive breast cancer patients: a real-world study. BMC Cancer. 2023;23:615.

[19] Terra Branco M, de Araujo Brito Buttros D, Carvalho-Pessoa E, Lima Sobreira M, Yukie Nakano Schincariol C, Nahas-Neto J, Nahas EAP. Atherosclerotic disease and cardiovascular risk factors in postmenopausal breast cancer survivors: a case-control study. Climacteric. 2019;22:202-7.

[20] Thomson CA, Thompson PA, Wright-Bea J, Nardi E, Frey GR, Stopeck A. Metabolic syndrome and elevated C-reactive protein in breast cancer survivors on adjuvant hormone therapy. J Womens Health (Larchmt). 2009;18:2041-7.

[21] Wung SF, Hepworth JT, Sparenga D, Merkle CJ. Cardiovascular Disease Risk and Breast Cancer Outcomes: A Pilot Study. Oncol Nurs Forum. 2015;42:E330-8.

[22] Yoon HJ, Kim HN, Yun Y, Kim Y, Ha AN, Kim HL, Kim BS. Background Intestinal 18F-FDG Uptake Is Related to Serum Lipid Profile and Obesity in Breast Cancer Patients. PLoS One. 2015;10:e0141473.

[23] Zhou Z, Zhang Y, Li Y, Jiang C, Wu Y, Shang L, et al. Metabolic syndrome is a risk factor for breast cancer patients receiving neoadjuvant chemotherapy: A case-control study. Front Oncol. 2022;12:1080054.
