# Supplementary material for: Association Between Triglyceride‐Glucose Index and Breast Cancer: A Systematic Review and Meta‐Analysis
Source: Cancer Rep (Hoboken). 2025 Apr 7;8(4):e70194. doi: 10.1002/cnr2.70194 (PMC11976027; doi:10.1002/cnr2.70194)
Supplement: Supplementary file 2 — Appendix S2 [file CNR2-8-e70194-s002.docx]

| **Study** | **Selection (0-4)** | **Comparability (0-2)** | **Outcome/Exposure (0-3)** | **Total (0-9)** |
| --- | --- | --- | --- | --- |
| Alkurt et al., 2022 | 3 | 1 | 2 | 6 |
| Da Silva et al., 2022 | 3 | 1 | 2 | 6 |
| Fritz et al., 2020 | 3 | 1 | 2 | 6 |
| Karadag et al., 2023 | 3 | 1 | 2 | 6 |
| Onder et al., 2024 | 3 | 2 | 2 | 7 |
| Panigoro et al., 2021 | 3 | 1 | 2 | 6 |
| Rachman et al., 2023 | 3 | 1 | 2 | 6 |
| Rajakumar et al., 2024 | 2 | 1 | 2 | 5 |
| Shi et al., 2022 | 3 | 1 | 2 | 6 |
| Wu et al., 2024 | 3 | 1 | 2 | 6 |
| Zhang et al., 2024 | 3 | 1 | 2 | 6 |
| Liu et al, 2024 | 3 | 2 | 3 | 8 |
| Li et al, 2024 | 3 | 1 | 2 | 6 |
