# Supplementary material for: Association Between Triglyceride‐Glucose Index and Breast Cancer: A Systematic Review and Meta‐Analysis
Source: Cancer Rep (Hoboken). 2025 Apr 7;8(4):e70194. doi: 10.1002/cnr2.70194 (PMC11976027; doi:10.1002/cnr2.70194)

**Supplementary Figures**

**Supplementary Figure 1.** Sensitivity analysis for the analysis comparing TyG index levels between patients with breast cancer and controls


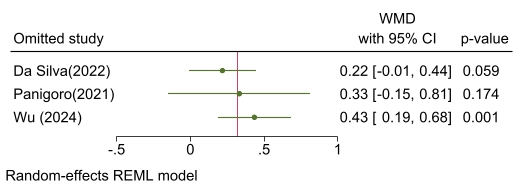


**Supplementary Figure 2.** Funnel plot assessing publication bias in studies comparing TyG index levels between breast cancer patients and controls.


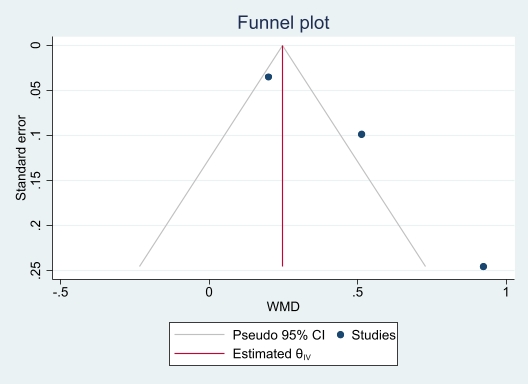


**Supplementary Figure 3.** Sensitivity analysis for cohort studies assessing the risk of breast cancer with increasing TyG index.


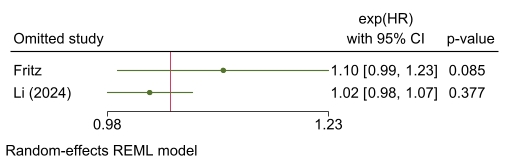


**Supplementary Figure 4.** Funnel plot assessing publication bias in cohort studies evaluating TyG index and breast cancer risk.


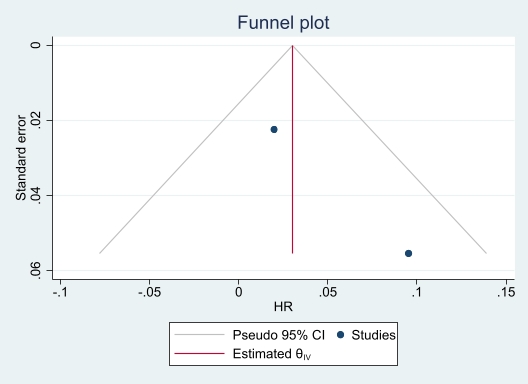


**Supplementary Figure 5.** Sensitivity analysis for the association between the highest TyG index group and breast cancer risk in cohort studies


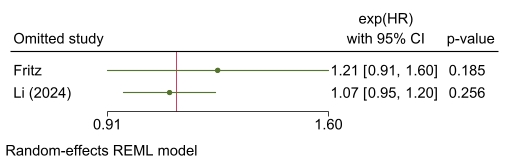


**Supplementary Figure 6.** Funnel plot assessing publication bias for studies analyzing the risk of breast cancer in the highest versus lowest TyG index groups in cohort studies


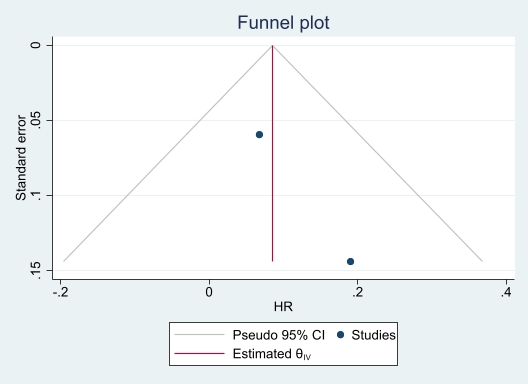


**Supplementary Figure 7.** Sensitivity analysis for the association between TyG index and breast cancer risk in case-control and cross-sectional studies.


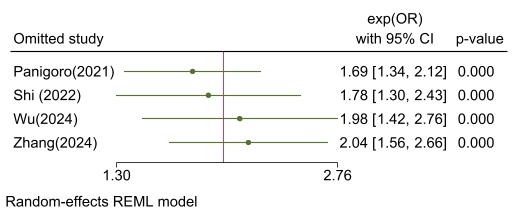


**Supplementary Figure 8.** Funnel plot assessing publication bias for case-control and cross-sectional studies on TyG index and breast cancer risk.


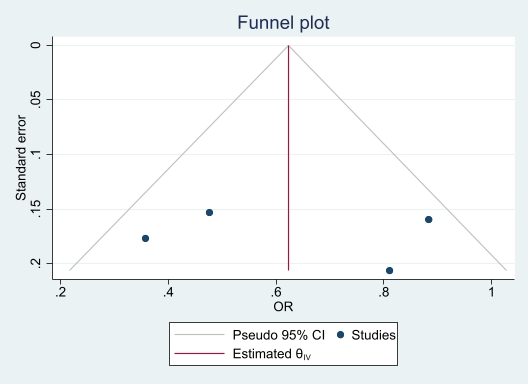


**Supplementary Figure 9.** Sensitivity analysis for the comparison of TyG index levels in patients with benign versus malignant breast lesions.


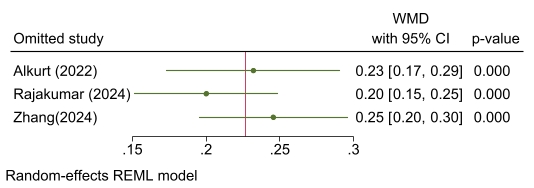


**Supplementary Figure 10.** Funnel plot assessing publication bias in studies comparing TyG index between malignant and benign breast lesions


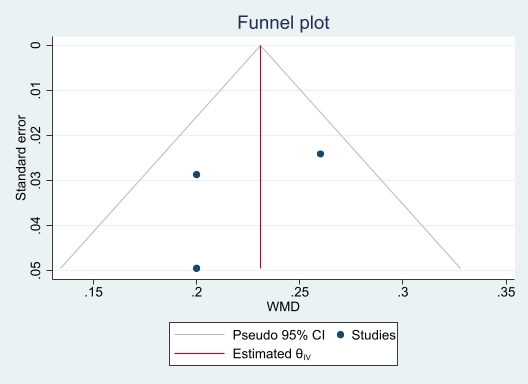

Supplement: Supplementary file 4 — Figures S1–S10 [file CNR2-8-e70194-s003.docx]
