# Supplementary material for: Association Between Triglyceride‐Glucose Index and Breast Cancer: A Systematic Review and Meta‐Analysis
Source: Cancer Rep (Hoboken). 2025 Apr 7;8(4):e70194. doi: 10.1002/cnr2.70194 (PMC11976027; doi:10.1002/cnr2.70194)
Supplement: Supplementary file 5 — Table S1 [file CNR2-8-e70194-s005.docx]

**Supplementary table 1: Search strategy**

| **Database** | **Code** | **Search formula** | **Findings** | **Last update** |
| --- | --- | --- | --- | --- |
| Pubmed | #1 | ("Breast Neoplasms"[Mesh] OR "Carcinoma, Ductal, Breast"[Mesh] OR “breast neoplasm”[title/abstract] OR “breast tumor” [title/abstract] OR “breast cancer” [title/abstract] OR “breast carcinoma” [title/abstract] OR “cancer of breast” [title/abstract] OR “cancer of the breast” [title/abstract] OR “malignant neoplasm of breast” [title/abstract] OR “breast malignant neoplasm” [title/abstract] OR “malignant tumor of breast” [title/abstract] OR “breast malignant tumor” [title/abstract] OR “mammary cancer” [title/abstract] OR “mammary carcinoma” [title/abstract] OR “mammary tumor” [title/abstract] OR “mammary neoplasm” [title/abstract] OR “human mammary neoplasm” [title/abstract] OR “human mammary carcinoma” [title/abstract] OR “invasive ductal carcinoma” [title/abstract] OR “mammary ductal carcinoma” [title/abstract] OR “infiltrating ductal carcinoma” [title/abstract]) | 493474 | (1/24/2025) |
|  | #2 | (“triglyceride glucose” [title/abstract] OR “triglyceride-glucose” [title/abstract] OR “lipid index” [title/abstract] OR “TyG” [title/abstract] OR “TyG index” [title/abstract] OR “triglyceride-glucose index” [title/abstract] OR “triglyceride glucose index” [title/abstract] OR “triglyceride and glucose index” [title/abstract] OR “triglyceride/glucose index” [title/abstract] OR “triacylglycerol glucose index” [title/abstract] OR “fasting glucose and triglyceride” [title/abstract] OR “fasting plasma glucose and triglyceride” [title/abstract] OR “fasting plasma glucose and triglyceride index” [title/abstract] OR “fasting triglyceride-glucose index” [title/abstract] OR “metabolic syndrome” [title/abstract] OR “syndrome X” [title/abstract] OR “Dysmetabolic Syndrome” [title/abstract] OR “Metabolic Cardiovascular Syndrome” [title/abstract] OR “Cardiovascular Syndrome” [title/abstract] OR “Cardiometabolic Syndrome” [title/abstract] OR “cardio metabolic risk factor” [title/abstract] OR “cardio-metabolic risk factor” [title/abstract] OR “insulin resistance” [title/abstract] OR "TyG-BMI"[Title/Abstract] OR "TyG Body Mass Index"[Title/Abstract] OR "Triglyceride-Glucose BMI"[Title/Abstract] OR "Triglyceride-Glucose Body Mass Index"[Title/Abstract] OR ("TyG"[title/abstract] and "BMI"[Title/Abstract]) OR "TyG-WC"[Title/Abstract] OR "TyG Waist Circumference"[Title/Abstract] OR "Triglyceride-Glucose WC"[Title/Abstract] OR "Triglyceride-Glucose Waist Circumference"[Title/Abstract] OR ("TyG"[title/abstract] and "WC"[Title/Abstract]) OR "TyG-WHtR"[Title/Abstract] OR "TyG Waist-to-Height Ratio"[Title/Abstract] OR "Triglyceride-Glucose WHtR"[Title/Abstract] OR "Triglyceride-Glucose Waist-to-Height Ratio"[Title/Abstract] OR ("TyG"[title/abstract] and "WHtR"[Title/Abstract])) | 164304 | (1/24/2025) |
|  | #3 | #1 AND #2 | 1033 | (1/24/2025) |
| Scopus | #4 | (TITLE-ABS-KEY("breast neoplasm") OR TITLE-ABS-KEY("breast tumor") OR TITLE-ABS-KEY("breast cancer") OR TITLE-ABS-KEY("breast carcinoma") OR TITLE-ABS-KEy("cancer of breast") OR TITLE-ABS-KEY("cancer of the breast") OR TITLE-ABS-KEY("malignant neoplasm of breast") OR TITLE-ABS-KEY("breast malignant neoplasm") OR TITLE-ABS-KEY("malignant tumor of breast") OR TITLE-ABS-KEY("breast malignant tumor") OR TITLE-ABS-KEY("mammary cancer") OR TITLE-ABS-KEY("mammary carcinoma") OR TITLE-ABS-KEY("mammary tumor") OR TITLE-ABS-KEY("mammary neoplasm”) OR TITLE-ABS-KEY("human mammary neoplasm”) OR TITLE-ABS-KEY("human mammary carcinoma") OR TITLE-ABS-KEY("invasive ductal carcinoma") OR TITLE-ABS-KEY("mammary ductal carcinoma") OR TITLE-ABS-KEY("infiltrating ductal carcinoma")) | 722,164 | (1/24/2025) |
|  | #5 | ( TITLE-ABS-KEY("triglyceride glucose") OR TITLE-ABS-KEY("triglyceride-glucose") OR TITLE-ABS-KEY("lipid index") OR TITLE-ABS-KEY("TyG") OR TITLE-ABS-KEY("TyG index") OR TITLE-ABS-KEY("triglyceride-glucose index") OR TITLE-ABS-KEY("triglyceride glucose index") OR TITLE-ABS-KEY("triglyceride and glucose index") OR TITLE-ABS-KEY("triglyceride/glucose index") OR TITLE-ABS-KEY("triacylglycerol glucose index") OR TITLE-ABS-KEY("fasting glucose and triglyceride") OR TITLE-ABS-KEY("fasting plasma glucose and triglyceride") OR TITLE-ABS-KEY("fasting plasma glucose and triglyceride index") OR TITLE-ABS-KEY("fasting triglyceride-glucose index") OR TITLE-ABS-KEY(“metabolic syndrome”) OR TITLE-ABS-KEY(“syndrome X”) OR TITLE-ABS-KEY(“Dysmetabolic Syndrome”) OR TITLE-ABS-KEY(“Metabolic Cardiovascular Syndrome”) OR TITLE-ABS-KEY(“Cardiovascular Syndrome”) OR TITLE-ABS-KEY(“Cardiometabolic Syndrome”) OR TITLE-ABS-KEY(“cardio metabolic risk factor”) OR TITLE-ABS-KEY(“cardio-metabolic risk factor”) OR TITLE-ABS-KEY(“insulin resistance”) OR TITLE-ABS-KEY ( "TyG-BMI" ) OR TITLE-ABS-KEY ( "TyG Body Mass Index" ) OR TITLE-ABS-KEY ( "Triglyceride-Glucose BMI" ) OR TITLE-ABS-KEY ( "Triglyceride-Glucose Body Mass Index" ) OR TITLE-ABS-KEY ( "TyG-WC" ) OR TITLE-ABS-KEY ( "TyG Waist Circumference" ) OR TITLE-ABS-KEY ( "Triglyceride-Glucose WC" ) OR TITLE-ABS-KEY ( "Triglyceride-Glucose Waist Circumference" ) OR TITLE-ABS-KEY ( "TyG-WHtR" ) OR TITLE-ABS-KEY ( "TyG Waist-to-Height Ratio" ) OR TITLE-ABS-KEY ( "Triglyceride-Glucose WHtR" ) OR TITLE-ABS-KEY ( "Triglyceride-Glucose Waist-to-Height Ratio" ) ) | 262,231 | (1/24/2025) |
|  | #6 | #4 AND #5 | 2016 | (1/24/2025) |
| Web of Science | #7 | TS=("triglyceride glucose" OR "triglyceride-glucose" OR "lipid index" OR "TyG" OR "TyG index" OR "triglyceride-glucose index" OR "triglyceride glucose index" OR "triglyceride and glucose index" OR "triglyceride/glucose index" OR "triacylglycerol glucose index" OR "fasting glucose and triglyceride" OR "fasting plasma glucose and triglyceride" OR "fasting plasma glucose and triglyceride index" OR "fasting triglyceride-glucose index" OR "metabolic syndrome" OR "syndrome X" OR "Dysmetabolic Syndrome" OR "human mammary carcinoma" OR "mammary ductal carcinoma" OR "Metabolic Cardiovascular Syndrome" OR "Cardiovascular Syndrome" OR "Cardiometabolic Syndrome" OR "cardio metabolic risk factor" OR "cardio-metabolic risk factor" OR "insulin resistance" OR "TyG-BMI" OR "TyG Body Mass Index" OR "Triglyceride-Glucose BMI" OR "Triglyceride-Glucose Body Mass Index" OR "TyG-WC" OR "TyG Waist Circumference" OR "Triglyceride-Glucose WC" OR "Triglyceride-Glucose Waist Circumference" OR "TyG-WHtR" OR "TyG Waist-to-Height Ratio" OR "Triglyceride-Glucose WHtR" OR "Triglyceride-Glucose Waist-to-Height Ratio") | 2920 | (1/24/2025) |
|  | #8 | TS=("breast neoplasm" OR "breast tumor" OR "breast cancer" OR "breast carcinoma" OR "cancer of breast" OR "cancer of the breast" OR "malignant neoplasm of breast" OR "breast malignant neoplasm" OR "malignant tumor of breast" OR "breast malignant tumor" OR "invasive ductal carcinoma" OR "infiltrating ductal carcinoma" OR "mammary cancer" OR "mammary carcinoma" OR "mammary tumor" OR "mammary neoplasm" OR "human mammary neoplasm" OR "human mammary carcinoma" OR "mammary ductal carcinoma") | 691221 | (1/24/2025) |
|  | #9 | #7 AND #8 | 2965 | (1/24/2025) |
| Embase | #10 | ('breast neoplasms' OR 'carcinoma, ductal, breast' OR 'breast neoplasm':ab,ti OR 'breast tumor':ab,ti OR 'breast cancer':ab,ti OR 'breast carcinoma':ab,ti OR 'cancer of breast':ab,ti OR 'cancer of the breast':ab,ti OR 'malignant neoplasm of breast':ab,ti OR 'breast malignant neoplasm':ab,ti OR 'malignant tumor of breast':ab,ti OR 'breast malignant tumor':ab,ti OR 'mammary cancer':ab,ti OR 'mammary carcinoma':ab,ti OR 'mammary tumor':ab,ti OR 'mammary neoplasm':ab,ti OR 'human mammary neoplasm':ab,ti OR 'human mammary carcinoma':ab,ti OR 'invasive ductal carcinoma':ab,ti OR 'mammary ductal carcinoma':ab,ti OR 'infiltrating ductal carcinoma':ab,ti) | 580203 | (1/24/2025) |
|  | #11 | ('triglyceride glucose':ab,ti OR 'triglyceride-glucose':ab,ti OR 'lipid index':ab,ti OR 'TyG':ab,ti OR 'TyG index':ab,ti OR 'triglyceride-glucose index':ab,ti OR 'triglyceride glucose index':ab,ti OR 'triglyceride and glucose index':ab,ti OR 'triglyceride/glucose index':ab,ti OR 'triacylglycerol glucose index':ab,ti OR 'fasting glucose and triglyceride':ab,ti OR 'fasting plasma glucose and triglyceride':ab,ti OR 'fasting plasma glucose and triglyceride index':ab,ti OR 'fasting triglyceride-glucose index':ab,ti OR 'metabolic syndrome':ab,ti OR 'syndrome x':ab,ti OR 'dysmetabolic syndrome':ab,ti OR 'metabolic cardiovascular syndrome':ab,ti OR 'cardiovascular syndrome':ab,ti OR 'cardiometabolic syndrome':ab,ti OR 'cardio metabolic risk factor':ab,ti OR 'cardio-metabolic risk factor':ab,ti OR 'insulin resistance':ab,ti OR 'TyG-BMI':ab,ti OR 'TyG body mass index':ab,ti OR 'triglyceride-glucose BMI':ab,ti OR 'triglyceride-glucose body mass index':ab,ti OR ('TyG':ab,ti AND 'BMI':ab,ti) OR 'TyG-WC':ab,ti OR 'TyG waist circumference':ab,ti OR 'triglyceride-glucose WC':ab,ti OR 'triglyceride-glucose waist circumference':ab,ti OR ('TyG':ab,ti AND 'WC':ab,ti) OR 'TyG-WHtR':ab,ti OR 'TyG waist-to-height ratio':ab,ti OR 'triglyceride-glucose WHtR':ab,ti OR 'triglyceride-glucose waist-to-height ratio':ab,ti OR ('TyG':ab,ti AND 'WHtR':ab,ti)) | 234897 | (1/24/2025) |
|  | #12 | #10 and #11 | 1617 | (1/24/2025) |
